# Mining Microbial Transcriptomes to Engineer Cell-Based Bacterial Biosensors in Gut-Resident Bacteroidaceae

**DOI:** 10.64898/2026.08.10.744002

**Published:** 2026-08-11

**Authors:** Joshua Glazier, David Villegas, Sandra McClure, Joyce Ghali, Jay Fuerte-Stone, Mark Mimee

## Abstract

The gastrointestinal tract is rich in metabolic, immune, and microbiome-derived signals that can inform the design of live biotherapeutics and diagnosis of intestinal disorders. Engineered cell-based biosensors can tap into this molecular information and report on their environment, yet their development in gut-resident symbionts has been limited by a lack of validated sensor systems. Here, we present a generalizable pipeline that leverages bacterial transcriptional profiling to identify environment-responsive systems for biosensor engineering. Candidate Sensors Systems (CSSs) mined from healthy, disease, and *in vitro* transcriptomes were assembled into a barcoded library in Bacteroidaceae chassis and screened in high-throughput *in vivo* to identify responsive promoters. A unique Bacteroidales ECF-type sigma factor operon with ties to sphingolipid metabolism and flux was highly responsive in chemically-induced colitis models. The biosensor responded robustly to disease and returned to baseline upon recovery, establishing an *in vivo* -driven strategy for discovering functional biosensors in non-model gut-resident bacteria.

## Full Text Availability

The license terms selected by the author(s) for this preprint version do not permit archiving in PMC. The full text is available from the preprint server.

